# Systemic Therapy for Metastatic Hormone-Sensitive Prostate Cancer: A Randomized Controlled Trial-Based Network Meta-Analysis

**DOI:** 10.1155/2022/7711555

**Published:** 2022-06-29

**Authors:** Zhen Kang, Wei Li, Yanhong Yu, Junfeng Yang

**Affiliations:** ^1^College of Medicine, Kunming University of Science and Technology, Kunming, Yunnan 650000, China; ^2^Department of Urology, The First People's Hospital of Yunnan Province, Kunming, Yunnan 650000, China

## Abstract

**Objective:**

To compare the effects of different treatment strategies for metastatic hormone-sensitive prostate cancer (mHSPC) using a network meta-analysis.

**Methods:**

English databases (PubMed, Embase, and medRxiv) and Chinese databases (Wanfang and CNKI) were searched for randomized controlled trials (RCTs) on the treatment of mHSPC from inception to June 1, 2021. The overall survival (OS) and failure-free survival (FFS) reported by the included studies were extracted from each study for network meta-analysis. Moreover, the priority ranks of the treatment methods were determined.

**Results:**

A total of 18 RCTs with 14,682 patients were included in this study. Androgen-deprivation therapy (ADT) + apalutamide (APA) showed the highest probability of improving the OS (96.2%) and FFS (68.0%). In addition, the patients were stratified into ten subgroups as follows: low/high tumor burden (CHAARTED criteria); Gleason score ≤7/≥8; Eastern Cooperative Oncology Group (ECOG) = 0/≥1; with/without prechemotherapy; and cooperative with/without concomitant radiotherapy. For the improvement of OS, the leading treatments were as follows: (1) ADT + enzalutamide (ENZA) (64.1%)/ADT + abiraterone acetate + prednisone (AAP) (54.3%); (2) ADT + ENZA (41.9%)/ADT + APA (39.2%); (3) ADT + ENZA (39.2%)/ADT + APA (32.1%); (4) ADT + radiotherapy (51%)/ADT + ENZA (76.7%); (5) ADT + AAP (51%)/ADT + AAP (60%).

**Conclusion:**

Three endocrine therapy drugs, abiraterone, enzalutamide, and APA, exhibited the best effects in improving the OS and FFS in all patients and subgroups; however, APA had the most prominent treatment effects. Therefore, ADT + APA should be applied as the common treatment for patients with HSPC based on objective and clinical conditions. *Trial Registration*. This meta-analysis has been registered on the PROSPERO website (Trial number: https://www.crd.york.ac.uk/prospero/CRD42020221062).

## 1. Introduction

Prostate cancer (PC) is the second most common cancer in men worldwide (annual age-standardized incidence of 29.3/100,000 men) and the most common cancer in the USA (annual age-adjusted incidence of 109.8/100,000 men) [1–4]. It commonly affects men ≥65 years old [5]. The major risk factors are older age, African or Caribbean descent, and family history of PC [1]. In the pathogenesis and progression of PC, patients always experience the transformation of the localized state into the metastatic state and the progress from the hormone-sensitive phase to the hormone-resistant phase. Various conditions require different types of treatments and prognostic monitoring, and the survival rate changes depending on the circumstances [6]. Typically, localized and locoregional PC has a good prognosis, with >99% of 5-year survival in the USA [2]. However, the prognosis of metastatic castration-resistant PC (mCRPC) is poor with a shorter survival time of 2- 3 years [7]. In localized PC, the preferred treatment method is radical prostatectomy with lymph node resection [6]. Depending on the risk level, radiotherapy, chemotherapy, and hormonotherapy are adopted after surgical removal. Further, the PSA change after operation must be strictly monitored. Combined with imaging techniques, such as PET/CT and multiparametric-MRI [8], the relapse of localized PC can be monitored. Different treatment strategies should be adopted if the condition tends to progress.

PC is an androgen-dependent tumor [9]. Therefore, the growth of tumors can be effectively controlled by inhibiting the binding of androgen receptors to the ligand, androgen. Generally, ADT is the fundamental remedy, and androgen receptor inhibitors are also incorporated in the treatment plan along with other treatment interventions. Currently, the most common treatment strategy uses one ADT (such as goserelin and degarelix) in combination with one endocrine therapy drug (such as abiraterone + prednisone (AAP), enzalutamide (ENZA), and apalutamide (APA)) [1,4,10]. Based on the disease and financial conditions of the patients, other treatments, including bicalutamide (BIC), bisphosphonate (BPs), and chemoradiotherapy, are also used in clinical practice.

During the treatment, a significant number of patients exhibit drug resistance. And patients are no longer sensitive to androgen blockers, and the disease progresses to the CRPC phase. Furthermore, only a few drugs are available, and the drug efficacy is diminishing. Subsequently, the mortality rate increases in PC [11]. Therefore, sustaining the hormone-sensitive phase is critical to prevent the disease progression. It is of great significance to initiate treatment intervention at the early stage of mHSPC. However, data about the impact of different strategies on the survival of patients with mHSPC are yet lacking.

## 2. Objective

Therefore, the present study aimed to compare the effects of different treatment methods on the overall survival (OS) and failure-free survival (FFS) of patients with mHSPC through direct meta-analysis and network meta-analysis based on previous relevant meta-analyses [12–14] and various randomized controlled trials (RCTs [15–32]). These findings provide comprehensive and accurate evidence for the clinical treatment of mHSPC.

## 3. Methods

### 3.1. Literature Search

This study was based on the published studies and trials, and no ethical approval or informed consent was required. It was conducted in agreement with the Declaration of Helsinki. The inclusion criteria were as follows: study type: RCT; population: mHSPC patients receiving systemic treatment; interventional methods: various treatment methods including endocrine therapy, chemotherapy, radiotherapy, and routine drug therapy; treatment in the control group: only ADT or other treatments; outcome measurements: OS and progression-free survival (PFS) summarized as hazard ratios (HRs) with 95% confidence interval (CI); and data report: studies reporting data of the outcomes. The following studies were excluded: repeated studies or duplicates, studies that only reported the efficacy of a systemic treatment method but did not compare the efficacies between two treatments, ongoing studies that only have interim data but no final analysis, and studies that confirmed the progression of mHSPC to CRPC.

### 3.2. Search Strategy

The English databases, PubMed, Embase, and medRxiv, and the Chinese databases, Wanfang and CNKI, were searched from their inception to June 1, 2021. The terms used for the search included prostate cancer; androgen-deprivation therapy; abiraterone; docetaxel (Doc); apalutamide; radiotherapy; bisphosphonate + celecoxib; enzalutamide; enzalutamide + Doc; bisphosphonate + Doc; celecoxib; bisphosphonate; bicalutamide; flutamide; metastatic hormone-sensitive prostate cancer; and the Chinese terms for metastatic hormone-sensitive prostate cancer, prostate cancer, androgen-deprivation therapy, enzalutamide, abiraterone, Doc, radiotherapy, bisphosphonate, bicalutamide, celecoxib, and flutamide.

### 3.3. Data Extraction

The literature search was performed by two investigators (Maolin Yang and Junfeng Yang) independently and in parallel. The records from the databases were input into EndNote X7 (Clarivate, Philadelphia, PA, USA). The studies were screened independently according to the predefined inclusion and exclusion criteria, and the full texts that met or possibly fulfilled the inclusion criteria were retrieved for further screening. Any disagreements during the screening were resolved by discussion with a third investigator.

For the screened studies, a predesigned Excel form was used to extract general information and outcome data. The general information included the following: (1) trial name, serial number, and authors' names; (2) follow-up time; (3) number, sex, and follow-up duration of the patients; (4) health scores of the patients (Eastern Cooperative Oncology Group, ECOG); (5) Gleason score; (6) proportions of patients with different tumor burdens; (7) median prostate-specific antigen (PSA) level of patients; (8) treatments in the study and control groups. The outcome data included the HR and the corresponding 95% CI of OS and FFS. For the studies that did not directly report the data, the 1745-6215-8-16-S1.xls worksheet was downloaded for secondary calculation [33].

### 3.4. Quality Assessment

The quality of the studies was assessed by two investigators independently using the Cochrane Risk of Bias Assessment Tool. The possible biases of the studies were assessed from five aspects, namely, selection bias (random sequence selection and allocation concealment), performance bias (blinding for participants and personnel), detection bias (blinding of outcome assessment), attrition bias (incomplete outcome data), and reporting bias (selective reporting). Each item was assessed according to the contents of the studies and classified as low bias, unknown, and high bias. The quality was assessed according to the Cochrane Handbook for Systematic Reviews [34].

### 3.5. Analysis Methods

The network meta-analysis was performed using the “gemtc” package (version 1.0-1) of R (version 4.0.5). A heterogeneity test was performed on the various direct control groups to calculate the *I*^2^ value. Then, the consistency of the direct control groups of different studies was evaluated based on the *I*^2^ value, and the distribution of the study effect was estimated by a forest plot. The present study used default parameters of Bayesian network meta-analysis and hypothesized the presence of common heterogeneity parameters in the network. In order to ensure the convergence of the model, the number of iterations was set at 100,000, of which the initial number was 20,000; the potential scale reduction factor (PSRF) evaluated the convergence. PSRF >1.05 indicated that the simulation times could not achieve high convergence, thereby requiring additional simulations. When the PSRF was <1.05 and close to 1, the convergence of the model was high. When the network meta-analysis was in a closed loop, the command “mtc.nodesplit” was used to evaluate the network inconsistency of the closed loop. *P*_inconsistency_ > 0.05 indicated the absence of inconsistency between the direct and indirect evidence in the closed loop. Network meta-analysis was conducted for the Bayesian fixed-effect model. The deviance information criterion (DIC) was used to evaluate the fitness of the fixed-effects and random-effects models, wherein a low DIC value indicated high model fitness. In this meta-analysis, only the findings of the model with the lowest DIC value were reported.

## 4. Results

### 4.1. Study Selection

**Figure 1:**
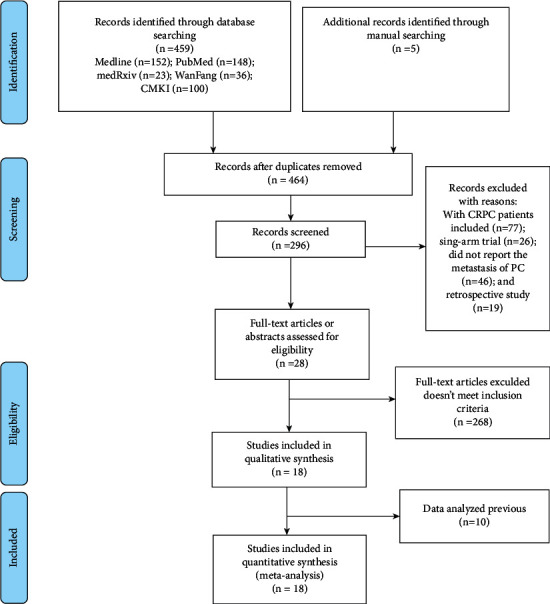
Literature search process.

A total of 464 studies were retrieved from the databases, according to the predefined search strategy and data collection method. After 263 duplicates were removed, the titles and abstracts of the remaining studies were reviewed, and 168 were excluded because the study subjects or interventional methods did not fulfill the inclusion criteria. Then, the full texts of the remaining 28 studies were downloaded and screened according to the inclusion criteria and completeness of data. Finally, 18 relevant RCTs were included in this study. The screening process is shown in Figure 1.

### 4.2. Characteristics of the Included Studies

A total of 18 RCTs with 14,682 patients were included, in which 12 systemic treatments had been performed for PC. These treatments included endocrine therapy by AAP, APA, BIC, and ENZA in 8 studies; chemoradiotherapy in 7 studies; and other treatments in 3 studies. The longest and shortest median follow-up duration were 83 and 24 months, respectively, and the median age of the patients was 70 years. The definition of FFS and the treatment details are given in Table 1.

### 4.3. Quality Assessment of the Included Studies

**Figure 2:**
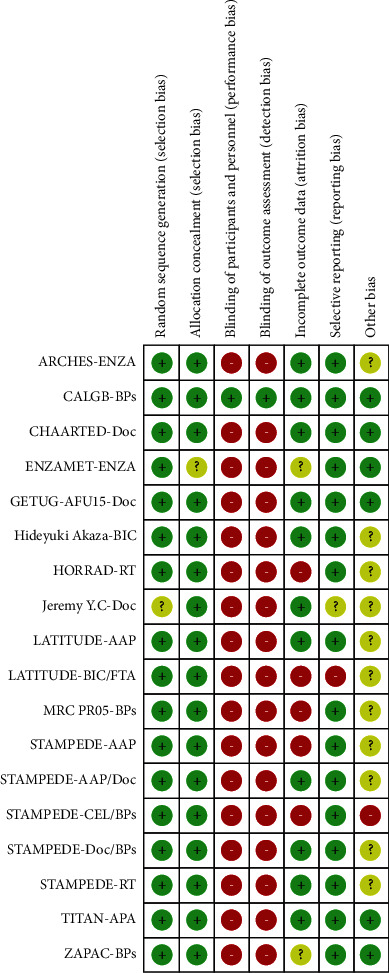
Evaluation of biases in the included studies. Green, low risk; red, high risk; yellow, unclear.

The quality of the included studies was assessed using the Cochrane Risk of Bias Assessment Tool. As shown in Figure 2, the risk of bias was acceptable in all the included studies.

### 4.4. Network Meta-Analysis

**Figure 3:**
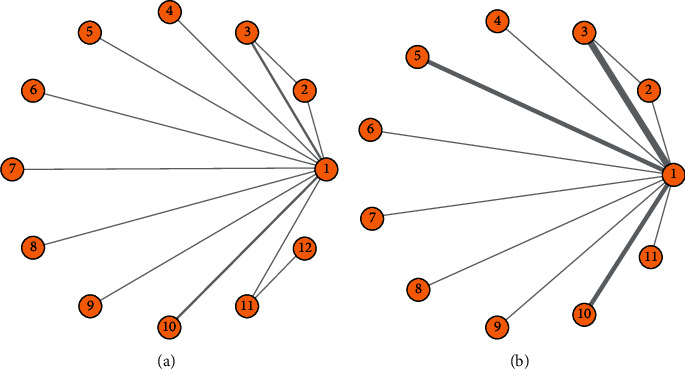
(a) Network graph of OS. (b) Network graph of FFS. 1, androgen-deprivation therapy; 2, ADT + abiraterone + prednisone; 3, ADT + docetaxel; 4, ADT + apalutamide; 5, ADT + radiotherapy; 6, ADT + bisphosphonate + celecoxib; 7, ADT + enzalutamide; 8, ADT + bisphosphonate + docetaxel; 9, ADT + celecoxib; 10, ADT + bisphosphonate; 11, ADT + bicalutamide; 12, ADT + flutamide.

**Figure 4:**
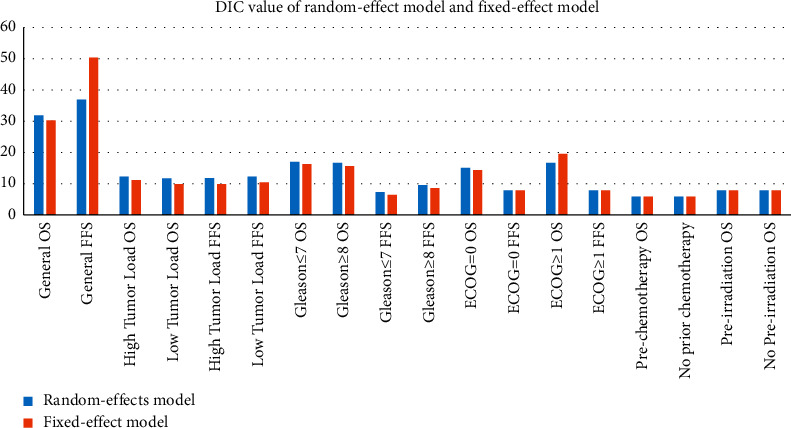
DIC values of different effect models.

The network graph of the various treatment methods is shown in Figure 3. Each circle represents one treatment method, the lines between the circles indicate the direct comparisons between treatment methods, and the other circles not linked by lines were compared indirectly. Simple ADT was used as the intermediate node for the indirect comparisons between each two of the included treatment methods. The network graph for OS and FFS of the patients included 12 and 10 treatment methods, respectively. All treatments except ADT + FTA were directly compared to ADT to assess the OS. For data analysis, this study simultaneously utilized the Bayesian random-effects and Bayesian fixed-effects models for network analysis, and DIC was used to evaluate the fitness. As shown in Figure 4, the DIC value of the random-effects model was low when evaluating the fitness of FFS of all the patients and the OS of the ECOG≥1 subgroup. The random-effects model was utilized for their pooled analysis, while the fixed-effects model was adopted for the other data.

### 4.5. Comparison of OS in All Patients

**Figure 5:**
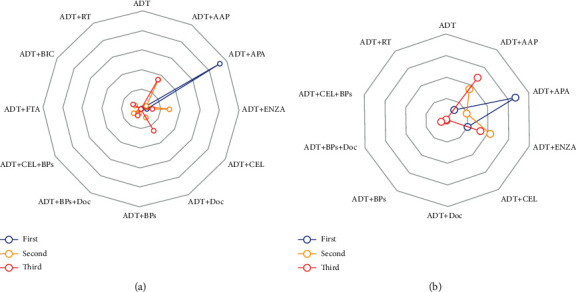
Radar plot of the first probabilities.

The network meta-analysis results are given in Table 2 (italics part). Except for ADT + RT, ADT + celecoxib (CEL), and ADT + flutamide (FTA), all combination treatment strategies showed significant advantages compared to the ADT treatment. As shown in Figure 5(a), the pairwise comparisons of the 12 treatment methods showed that ADT + APA is the most effective and, hence, the leading treatment (96.2%).

**Figure 6:**
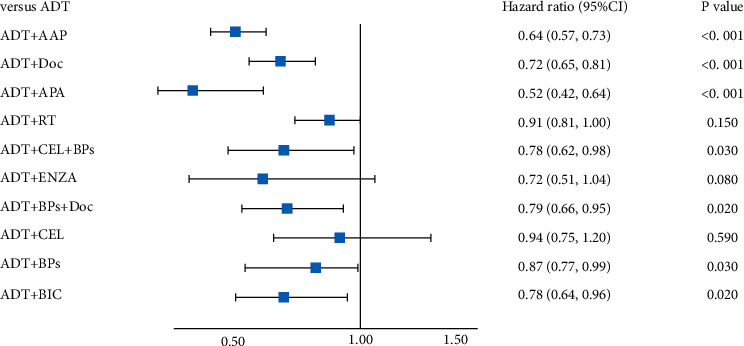
Forest plot of OS.

Figure 6 shows the results of the direct comparison of ADT with the combination of ADT and other treatments. Compared with ADT, the advantageous combination treatment methods are as follows: ADT + AAP (HR = 0.69, 95% CI = 0.62–0.78), ADT + Doc (HR = 0.72, 95% CI = 0.65–0.81), ADT + APA (HR = 0.52, 95% CI = 0.42–0.64), ADT + CEL + BPs (HR = 0.78, 95% CI = 0.62–0.98), ADT + ENZA (HR = 0.70, 95% CI = 0.57–0.87), ADT + Doc + BPs (HR = 0.79, 95% CI = 0.66–0.95), ADT + BPs (HR = 0.87, 95% CI = 0.77–0.99), and ADT + BIC (HR = 0.78, 95% CI = 0.64–0.96). And the other three methods—ADT + RT (HR = 0.92, 95% CI = 0.81–1.00), ADT + FTA (HR = 1.00, 95% CI = 0.78–1.40), and ADT + CEL (HR = 0.94, 95% CI = 0.75–1.20)—have no significant difference compared with ADT.

### 4.6. Comparison of FFS in All Patients

The network meta-analysis results are given in Table 2 (bold part). Except for ADT + BPs, ADT + CEL + BPs, and ADT + CEL, the other seven combination treatment strategies showed significant advantages in FFS compared to ADT treatment. As shown in Figure 5(b), the pairwise comparisons of the ten treatment methods showed that ADT + APA, ADT + AAP, and ADT + ENZA were the three most effective treatments with “first” possibility of 67.7%, 11.2%, and 20.2%, respectively.

**Figure 7:**
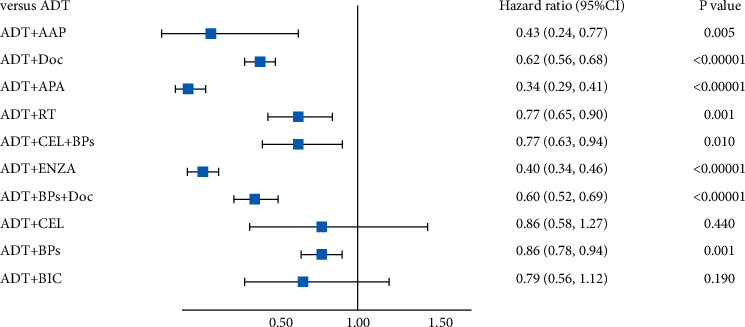
Forest plot of FFS.

Figure 7 shows the results of the direct comparison of ADT with the combination therapy by ADT and other treatments. Compared to ADT, the effects of the combination therapy were as follows: ADT + AAP (HR = 0.41, 95% CI = 0.32–0.52), ADT + ENZA (HR = 0.40, 95% CI = 0.29–0.54), ADT + APA (HR = 0.34, 95% CI = 0.23–0.51), ADT + Doc + BPs (HR = 0.60, 95% CI = 0.40–0.89), ADT + BPs (HR = 0.87, 95% CI = 0.84–1.00), ADT + CEL + BPs (HR = 0.77, 95% CI = 0.51–1.20), ADT + CEL (HR = 0.86, 95% CI = 0.57–1.30), ADT + RT (HR = 0.75, 95% CI = 0.57–0.96), and ADT + Doc (HR = 0.63, 95% CI = 0.51–0.77).

### 4.7. Subgroup Analysis

**Figure 8:**
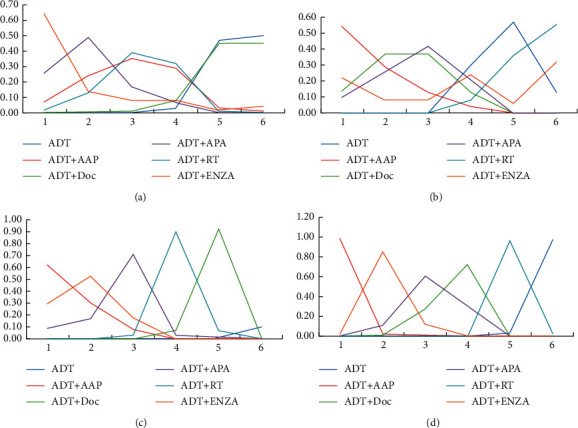
Line charts of the orders of suitable drugs for patients with different tumor burdens. (a) OS of patients with a low tumor burden. (b) OS of patients with a high tumor burden. (c) FFS of patients with a low tumor burden. (d) FFS of patients with a high tumor burden.

Low/high tumor burden subgroups: according to the CHAARTED criteria, PC patients with at least one visceral metastasis and/or more than four bone metastases and with at least one pelvic metastasis or extraspinal metastasis were classified into the high tumor burden subgroup, while the other patients comprised the low tumor burden subgroup. ADT + ENZA had the highest treatment effects (probability of 67.3%) in improving OS for the low tumor burden patients, while ADT + AAP showed maximal treatment effects (probability of 61.7%) in improving FFS. ADT + AAP had the highest treatment effects for the high tumor burden patients in improving both OS (probability of 66.1%) and FFS (probability of 97.6%). The order of effects of the other treatments is shown in Figure 8.

**Figure 9:**
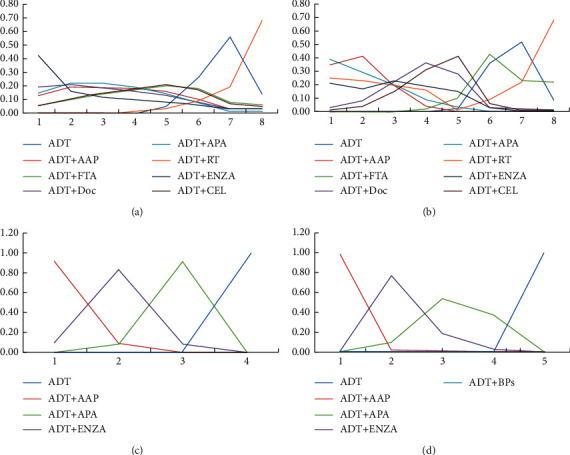
Line charts of the orders of suitable drugs for patients with different Gleason scores. (a) Gleason score ≤7 (OS). (b) Gleason score ≥8 (OS). (c) Gleason score ≤7 (FFS). (d) Gleason score ≥8 (FFS).

Gleason score ≤7/≥8 subgroups: for patients with a Gleason score of ≤7, ADT + ENZA had the highest treatment effects (probability of 41.9%) in improving the OS. Among the four treatments improving the FFS of patients, ADT + APA had the highest treatment effects (probability of 90.8%). For patients with a Gleason score of ≥8, ADT + APA had the highest treatment effects (probability of 39.2%), improving the OS among the eight methods. Furthermore, among the five treatments improving the FFS of the patients, ADT + AAP had maximal treatment effects (probability of 98.3%). The order of effects of the other treatments is shown in Figure 9.

**Figure 10:**
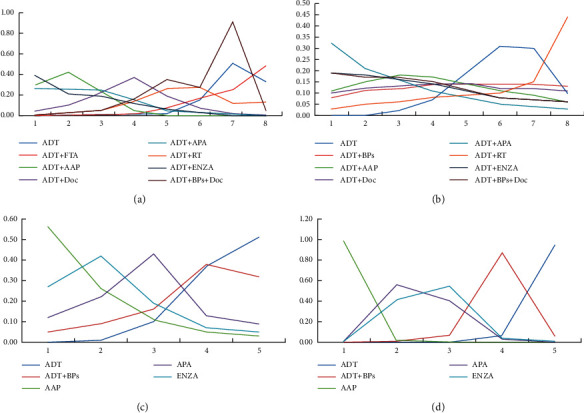
Line charts of the orders of suitable drugs for patients with different ECOG scores. (a) ECOG = 0 (OS). (b) ECOG ≥1 (OS). (c) ECOG = 0 (FFS). (d) ECOG ≥1 (FFS).

ECOG score 0/≥1 subgroups: for patients with an ECOG score of 0, ADT + ENZA had the highest treatment effects (probability of 39.2%) in improving the OS, and ADT + AAP had the highest treatment effects (probability of 27.2%) in improving the FFS. For patients with an ECOG score of ≥1, ADT + APA had the highest treatment effects (probability of 32.1%) in improving the OS, and ADT + AAP had the highest treatment effects (probability of 98.0%) in improving the FFS. The order of the effects of the other treatments is shown in Figure 10.

**Figure 11:**
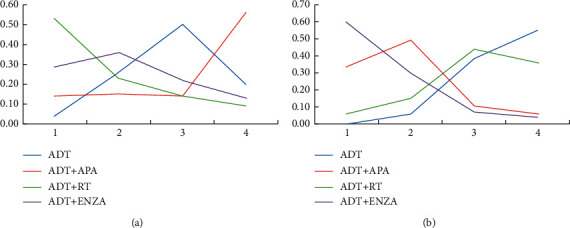
Line charts of the orders of suitable drugs for patients who received chemotherapy before. (a) Receiving previous chemotherapy (OS). (b) Not receiving previous chemotherapy (OS).

With/without chemotherapy (Doc) before systemic therapy subgroups: some of the patients had already been treated with six cycles of Doc (3 weeks, 75 mg/m^2^) before participating in the included RCTs. For such patients, the treatment effect of ADT + RT was maximal in improving the OS but not statistically significant (HR = 0.81, 95% CI = 0.49–1.34). For the patients who did not receive Doc treatment before, the treatment effect of ADT + ENZA was maximal in improving the OS (76.7%), indicating that the treatment efficacy of ENZA was not affected significantly by previous chemotherapy. The results of all the other treatments were not statistically significant, irrespective of the previous chemotherapy. The order of the treatments' effects is shown in Figure 11.

**Figure 12:**
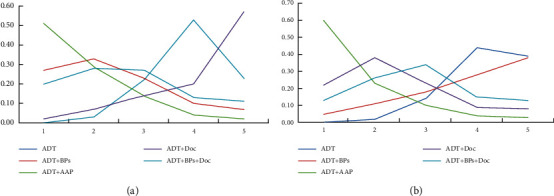
Line charts of the orders of suitable drugs for patients who received radiotherapy. (a) Receiving radiotherapy (OS). (b) Not receiving radiotherapy (OS).

Subgroups by radiotherapy: for patients who underwent radiotherapy within 6–9 months after randomization, ADT + AAP had the highest effects in improving OS (50.7%), but the result was not statistically significant (HR = 0.64, 95% CI = 0.38–1.08). For patients who did not receive radiotherapy within 6–9 months after randomization, AAP + ADT had the highest effects in improving OS (60%), indicating that the treatment effects of abiraterone were not significantly influenced by radiotherapy. The order of the effects of the other treatments is shown in Figure 12.

**Figure 13:**
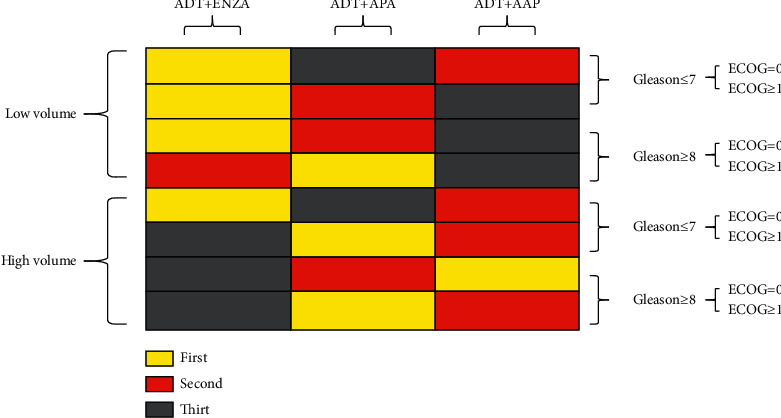
Probability ranks of the prior drugs for some subgroups.

Summary of subgroup analyses: the conditions of the patients are complex in clinical practice. Since the selection of drugs by different patients is an independent event, the method of multiplication of probability was adopted to identify the suitable drugs under diverse conditions (Figure 13). For instance, ADT + ENZA had the highest effects in improving the OS of patients with low tumor burden, Gleason score ≤7, and ECOG = 0. Therefore, ADT + ENZA should be administered if allowed by the objective conditions. Currently, only the Gleason score, ECOG score, and tumor burden are considered in the analysis, and the findings are only applicable for the decisions wherein no other influencing factors are present. In clinical practice, the most suitable drugs should be selected based on the disease conditions and demands of the patients.

## 5. Discussion

PC is one of the most common malignant tumors of the urinary system in male patients. The mHSPC phase of PC is “controllable,” but if the disease progresses to the CRPC phase, aggressive treatment only provides limited benefits to the patients. Therefore, early intervention in the mHSPC phase could maximize the survival benefits of patients. This meta-analysis aimed to compare the effects of different treatment strategies for mHSPC using a network meta-analysis. The results suggested that three endocrine therapy drugs, namely, AAP, ENZA, and APA, improve the OS and FFS in all patients and different subgroups. Interestingly, the treatment effects of APA are the most prominent. Therefore, ADT + APA should be applied as the common treatment for patients with HSPC.

Several previous network meta-analyses on drug therapy in mHSPC patients have already been published. The study by Chen [9] compared the effects of ADT in combination with novel endocrine therapy drugs. With the update of databases, we performed a comprehensive search of the literature, included 12 treatment methods for comparison, and performed subgroup analyses in ten subgroups. The results showed that compared to ADT monotherapy, the combinations of ADT with AAP, ENZA, or APA are the most effective treatments. The subgroup analysis was carried out to identify suitable treatment methods. The effects of ADT + AAP in improving the OS of patients with low tumor burden are slightly lower than those of ADT + ENZA and ADT + APA; however, with the increase in tumor burden, the effects of ENZA, an AR antagonist, decreased gradually, which confirmed that the effects of AR antagonists change with the progression of the disease. A previous study demonstrated that due to genetic alterations, about 15–20% of advanced CRPCs become independent of the AR signaling pathway [35]. Therefore, the progression from low tumor burden mHSPC to high tumor burden mHSPC and then to CRPC reflects the alterations in the *AR* gene. Various genetic variations of the possible targets were detected in PC samples [36], but no targeted therapy drug has shown clinical benefits, and the predictive biomarkers are also under investigation.

Previous studies have shown that AR abnormalities are associated with drug resistance [37,38]. For instance, the expression of AR-V7 RNA mutant is associated with resistance to abiraterone and enzalutamide. Several RCTs are currently ongoing to investigate the treatments for CRPC from the genetic aspect, such as using poly-ADP-ribose polymerase (PARP) inhibitors [39,40]. PC patients with BRCA 1/2 mutations may have a potential effect on PARP inhibitors. Compared with traditional hormonotherapy, PARP inhibitors can effectively improve the survival of patients with CRPC. However, the research studies on PARP are lacking for patients with HSPC.

Although immunological therapy elicits successful effects in various tumors, it barely shows satisfactory results in the treatment of PC. A total of 296 patients, including patients with mCRPC, malignant melanoma, nonsmall cell lung cancer, renal cancer, and colorectal cancer, participated in a phase IB trial of Nivolumab (PD-1 antibody). After being treated with 12 cycles of Nivolumab, 17 patients with mCRPC did not have objective remission. However, the objective remission rate for patients with nonsmall cell lung cancer, melanoma, and renal cancer was 18%, 28%, and 27% (NCT00730639), respectively [41]. According to a large, ongoing phase II clinical trial of Pembrolizumab in patients with mCRPC, the objective remission rate for Pembrolizumab treatment was only 3%–5% [42]. The results of two phase III trials on CTLA-4 immunosuppressant Ipilimumab in mCRPC were not optimistic as well. The experimental results failed to meet the primary endpoint of overall survival, although the progression-free survival extended to a certain degree [43, 44]. Based on the current research, the immune checkpoint inhibitor monotherapy is not effective in treating PC, and the specific mechanism for this ineffectiveness needs to be identified. Thus, further investigation is needed on the treatments from the genetic aspect in mHSPC patients. The genetic alterations or changes in the expression of RB1, BRCA1/2, ATM, CDK12, and PTEN have been reported to influence the OS and FFS in mHSPC and mCRPC [45]. These phenomena could be applied to select the treatment or eventually as treatment targets.

The subgroup analyses in this study showed that ADT + AAP and ADT + APA are the most effective drugs in improving the survival of patients under most conditions, while AAP had the advantage of delaying the disease progression in certain subgroups, indicating that it could delay the progression from the mHSPC phase to the CRPC phase. The effects of ADT + AAP in improving the OS of patients in the mHSPC phase were slightly lower than those of AR inhibitors, such as ADT + ENZA and ADT + APA. Nonetheless, the effects of ADT + AAP were improved in patient subgroups with severe disease, as assessed by both OS and FFS. The long-term STAMPEDE trial [15] also demonstrated that AAP is the most comprehensive androgen blocking drug with unique advantages. In addition, abiraterone is covered by medical insurance in China, and thus the cost of AAP treatment is lower than that of the other equivalent drugs. Therefore, various factors should be considered in clinical practice to select the optimal drugs. With an increasing number of drugs being covered by medical insurance, the treatment alternatives will continue to expand.

Regarding the matching of radiotherapy and chemotherapy, this study only compared the effects of several treatments on OS due to the limited number of included databases. Radiotherapy or chemotherapy alone could improve the survival of the patients, and the findings of this study showed that the combination of other treatments with either of these approaches did not result in significant survival benefits. Of course, the data origin included in our study were first treated with chemotherapy and then endocrine therapy. This model is not equivalent to the formal “combined chemotherapy” treatment, and the results are not representative, but it also triggered our thinking about whether the simultaneous use of endocrine drugs and chemotherapy can play a more ideal therapeutic role. Therefore, additional combined treatments should be discussed in clinical practice.

Nevertheless, the present study has several limitations. First, several studies did not describe the metastases clearly, leading to heterogeneity in the findings. However, most studies still grouped the patients. Second, the closed-loop consistency test showed inconsistencies in the closed-loop ADT-ADT + Doc-ADT. Although direct comparison and network analyses were performed simultaneously, the inconsistency could influence the results. Third, this study mainly compared the efficacy of drugs but did not compare the adverse events and patients' quality of life during treatments [46], which would be examined in the future. Finally, most of the studies used ADT as the control group, and the evidence from direct comparison is unavailable.

## 6. Conclusions

The endocrine therapy drugs, AAP, ENZA, and APA, have the best efficacy in improving the OS and FFS of patients with mHSPC and different subgroups; among them, APA exhibits the most prominent treatment effects. Therefore, ADT + APA should be selected as the common treatment for mHSPC in clinical practice.

## Figures and Tables

**Table 1:** Characteristics of the included studies.

Study	Time of the study	Study group	Control group	Sample size (study group/control group)	Median age (years)	Follow-up time (months)	WHO ECOG score, *n* (%)	Gleason score, *n* (%)	Tumor burden (high%/low%)	Median PSA level (ng/ml)	Definition of failure-free survival
STAMPEDE-AAP	From November 2011 to January 2014	ADT + AAP	ADT	1,917 (957/960)	67	40	0: study group, 746 (78%); control group, 749 (78%) 1 or 2: study group, 211 (22%); control group, 211 (22%)	≤7: study group, 223 (23%); control group, 221 (23%) 8 to 10: study group, 721 (75%); control group, 715 (74%) unknown: study group, 13 (1%); control group, 24 (2%)	45%/55%	53	Time from randomization to the occurrence of imaging progression, PSA progression, or death of patient

CHAARTED-Doc	From July 2006 to December 2012	ADT + Doc	ADT	790 (393/397)	64	53.7	Not recorded	Not recorded	65%/35%	Not recorded	Time from randomization to the progression to CRPC phase of the patient

TITAN-APA	From December 2015 to July 2020	ADT + APA	ADT	1,052 (527/525)	68	55	0: study group, 328 (62.5%); control group, 348 (66.0%) 1: study group, 197 (37.5%); control group, 178 (33.8%) 2: study group, 0 (0%); control group, 1 (0.2%)	<7: study group, 41 (7.8%); control group, 39 (7.4%) 7: study group, 133 (25.3%); control group, 130 (24.7%) >7: study group, 351 (66.9%); control group, 358 (67.9%)	63%/37%	Not recorded	Time from randomization to the progression to CRPC phase of the patient

STAMPEDE-RT	From January 22, 2013, to September 2, 2016	ADT + RT	ADT	2,061 (1,029/1,032)	68	Not recorded	0: study group, 732 (71%); control group, 734 (71%) 1-2: study group, 297 (29%); control group, 298 (29%)	≤7: study group, 173 (17%); control group, 172 (18%) 8–10: study group, 820 (83%); control group, 810 (82%) Unknown: study group, 36; control group, 50	54.3%/39.7%	98	Time from randomization to the occurrence of at least one of the following events: biochemical recurrence, local progression, lymph node metastasis or distal metastasis, and death of patient

HORRAD-RT	From 2004 to 2014	ADT + RT	ADT	532 (216/216)	67	47	0: study group, 187 (87%); control group, 176 (82%) 1: study group, 22 (10%); control group, 31 (14%) 2: study group, 4 (2%); control group, 6 (3%) 3: study group, 3 (1%); control group, 3 (1%)	6: study group, 7 (3%); control group, 7 (3%) 7: study group, 66 (31%); control group, 64 (30%) 8: study group, 48 (22%); control group, 65 (30%) 9: study group, 85 (39%); control group, 72 (33%) 10: study group, 9 (4%); control group, 7 (3%) Unknown: study group, 1 (1%); control group, 1 (1%)	Not recorded	142	Time from randomization to the occurrence of at least one of the following events: biochemical recurrence, local progression, lymph node metastasis or distal metastasis, and death of patient

LATITUDE-AAP	From February 12, 2013, to December 11, 2014	ADT + AAP	ADT	1,199 (597/602)	67	51.8	0-1: study group, 573 (47.8%); control group, 586 (48.9%) 2: study group, 24 (0.02%); control group, 16 (0.01%)	<7: study group, 4 (1%); control group, 1 (<1%) 7: study group, 9 (2%); control group, 15 (2%) ≥8: study group, 584 (98%); control group, 586 (97%)	Not recorded	Not recorded	Time from randomization to imaging-based progression

GETUG-AFU15-Doc	From October 2004 to December 2008	ADT + Doc	ADT	385 (192/193)	63.5	83.9	0: study group, 181 (99); control group, 176 (96%) 1-2: study group, 2 (1%); control group, 7 (4%)	<7: study group, 84 (45%); control group, 78 (41%) ≥8: study group, 103 (55%); control group, 113 (59%)	24.7%/75.3%	26.5	Time from randomization to imaging progression, PSA progression, or death of patient

STAMPEDE-Doc/BPs	From October 5, 2005 to March 31, 2013	ADT + DocADT + BPsADT + BPs + Doc	ADT	2,962 (592/593/593/1,184)	65	43	Not recorded	≤7: study group, Doc: 110 (19%); BPs: 122 (21%); BPs + Doc: 117 (20%); control group, 282 (24%) 8–10: study group, BPs: 421 (71%); Doc: 436 (74%); BPs + Doc: 425 (72%); control group, 810 (68%) Unknown: study group, BPs: 50 (8%); Doc: 46 (8%); BPs + Doc: 51 (9%); control group, 92 (8%)	Not recorded	Not recorded	Time from randomization to the occurrence of at least one of the following events: biochemical recurrence, local progression, lymph node metastasis or distal metastasis, and death of patient

ARCHES-ENZA	From August 2017 to October 14, 2018	ADT + ENZA	ADT	1,150 (574/576)	70	14.4	0: study group, 448 (78.0%); control group, 443 (76.9%) 1: study group, 125 (21.8%); control group, 133 (23.1%)	<8: study group, 171 (29.8%); control group, 187 (32.5%) ≥8: study group, 386 (67.2%); control group, 373 (64.8%)	63.2%/36.8%	5.2	Time from randomization to imaging progression or death of patient

STAMPEDE-CEL	From October 17, 2005, to January 31, 2011	ADT + CEL + BPs	ADT	567 (190/377)	65	69	Not recorded	Not recorded	Not recorded	62	Time from randomization to the occurrence of at least one of the following events: PSA progression, local progression, lymph node progression, progression of existing lesions or appearance of new metastatic lesions, and death of patient

STAMPEDE-CEL	From October 17, 2005, to January 31, 2011	ADT + CEL	ADT	873 (291/582)	65	69	0: study group, 233 (77%); control group, 448 (77%) 1: study group, 64 (22%); control group, 127 (22%) 2: study group, 4 (1%); control group, 8 (1%)	Not recorded	Not recorded	62	Time from randomization to the occurrence of at least one of the following events: PSA progression, local progression, lymph node progression, progression of existing lesions or appearance of new metastatic lesions, and death of patient

MRC PR05-BPs	From June 1994 to July 1998	ADT + BPs	ADT	311 (155/156)	71	59	0: study group, 101 (65%); control group, 103 (66%) 1: study group, 46 (30%); control group, 42 (27%) 2: study group, 8 (5%); control group, 11 (7%)	Not recorded	Not recorded	Not recorded	Time from randomization to bone metastasis (namely, requiring the start of further treatment) or death of patient

CALGB-BPs	From January 15, 2004, to May 31, 2012	ADT + BPs	ADT	642 (323/322)	66	59	0: study group, 205 (63%); control group, 205 (64%) 1: study group, 105 (33%); control group, 105 (33%) 2: study group, 13 (4%); control group, 12 (4%)	1: study group, 2 (<1%); control group, 3 (<1%) 5–7: study group, 122 (38%); control group, 112 (35%) 8–10: study group, 187 (58%); control group, 186 (58%) Unknown: study group, 12 (4%); control group, 21 (7%)	Not recorded	6.9	Time from randomization to bone metastasis, PSA progression, or death of patient

ZAPCA-BPs	From May 2008 to December 2010	ADT + BPs	ADT	227 (109/110)	72	41.5	0: study group, 74 (67.9%); control group, 77 (70.0%) 1: study group, 31 (28.4%); control group, 27 (24.5%) 2: study group, 4 (3.7%); control group, 6 (5.5%)	≤7: study group, 21 (19.3%); control group, 18 (16.4%) ≥8: study group, 88 (80.7%); control group, 92 (83.6%)	Not recorded	371	Time from randomization to clinical progression, PSA progression, death of any cause, or treatment discontinuation

Jeremy YC-Doc	From January 2015 to July 2016	ADT + Doc	ADT	64 (32/32)	65	Not recorded	Not recorded	6: study group (3.6%); control group (11.5%) 7: study group (10.7%); control group (7.7%) 8–10: study group (85.7%); control group (80.8%)	29/3	Not recorded	Time from randomization to the occurrence of at least one of the following events: PSA progression, clinical progression, and imaging progression

Hideyuki Akaza-BIC	From December 2003 to March 2007	ADT + BIC	ADT	205 (102/103)	75	62.4	Not recorded	Not recorded	Not recorded	Not recorded	Not recorded

LATITUDE-BIC/FTA	From January 1992 to September 1993	ADT + BIC	ADT + FTA	813 (404/409)	70	11.4	0: study group, 221 (55%); control group, 208 (51%) 1: study group, 143 (35%); control group, 150 (37%) 2: study group, 40 (10%); control group, 51 (13%)	Not recorded	Not recorded	Not recorded	Not recorded

ENZAMET -ENZA	From March 2014 to March 2020	ADT + ENZA	ADT	1,125 (563/562)	69	34	Not recorded	≤7: study group, 152 (27%); control group, 163 (29%) ≥8: study group, 335 (60%); control group, 321 (57%) Missing data: study group, 76 (13%); control group, 78 (14%)	52%/48%	Not recorded	Time from randomization to clinical progression or PSA progression of patient

STAMPEDE-AAP/Doc	From November 2011 to March 2013	ADT + AAP	ADT + Doc	566 (377/189)	66	48	0: study group, 300 (71%); control group, 149 (71%) 1-2: study group, 77 (29%); control group, 40 (29%)	≤7: study group, 21 (19.3%); control group, 18 (16.4%) ≥8: study group, 88 (80.7%); control group, 92 (83.6%)	Not recorded	56	Time from randomization to the occurrence of at least one of the following events: PSA progression, local progression, lymph node progression, progression of the existing lesions or appearance of new metastatic lesions, and death of patient

**Table 2:** League table of the overall survival (OS; italics) and failure-free survival (FFS; bold) comparison of treatment methods, showing the network meta-analysis results of the hazard ratios (HR) and corresponding 95% confidential intervals (95% CI).

ADT	0.41 (0.32, 0.52)	0.63 (0.51, (0.78)	0.34 (0.23, 0.5)	0.8 (0.61, 1.06)	0.77 (0.51, 1.13)	0.4 (0.29, 0.54)	0.6 (0.41, 0.89)	0.86 (0.57, 1.29)	0.84 (0.68, 1.03)	NA	NA
*1.45 (1.29, 1.63)*	ADT + AAP	**1.53 (1.17, 2.01)**	**0.82 (0.53, 1.32)**	**1.93 (1.38, 2.79)**	**1.86 (1.18, 3.02)**	**0.96 (0.66, 1.42)**	**1.45 (0.93, 2.32)**	**2.08 (1.31, 3.42)**	**2.03 (1.48, 2.79)**	NA	NA
*1.38 (1.24, 1.54)*	*0.95 (0.83, 1.09)*	ADT + Doc	**0.54 (0.34, 0.85)**	**1.27 (0.9, 1.81)**	**1.21 (0.78, 1.93)**	**0.63 (0.44, 0.91)**	**0.95 (0.62, 1.47)**	**1.36 (0.87, 2.18)**	**1.33 (0.99, 1.79)**	NA	NA
*1.92 (1.56, 2.37)*	*1.33 (1.05, 1.69)*	*1.39 (1.1, 1.77)*	ADT + APA	**2.34 (1.46, 3.86)**	**2.26 (1.29, 3.92)**	**1.17 (0.71, 1.93)**	**1.75 (1.01, 3.08)**	**2.53 (1.43, 4.4)**	**2.46 (1.58, 3.88)**	NA	NA
*1.09 (0.97, 1.23)*	*0.75 (0.64, 0.89)*	*0.79 (0.67, 0.93)*	*0.57 (0.45, 0.72)*	ADT + RT	**0.96 (0.59, 1.56)**	**0.5 (0.33, 0.74)**	**0.75 (0.46, 1.19)**	**1.07 (0.67, 1.75)**	**1.05 (0.74, 1.46)**	NA	NA
*1.28 (1.02, 1.61)*	*0.88 (0.68, 1.14)*	*0.93 (0.72, 1.2)*	*0.67 (0.49, 0.91)*	*1.17 (0.91, 1.52)*	ADT + CEL + BPs	**0.52 (0.32, 0.86)**	**0.78 (0.44, 1.37)**	**1.12 (0.65, 1.99)**	**1.09 (0.7, 1.73)**	NA	NA
*1.42 (1.15, 1.76)*	*0.98 (0.77, 1.25)*	*1.03 (0.80, 1.31)*	*0.74 (0.55, 1.00)*	*1.30 (1.02, 1.67)*	*1.11 (0.81, 1.52)*	ADT + ENZA	**1.12 (0.91, 2.46)**	**2.16 (1.32, 3.62)**	**2.11 (1.48, 3.04)**	NA	NA
*1.27 (1.05, 1.53)*	*0.87 (0.7, 1.09)*	*0.92 (0.74, 1.14)*	*0.66 (0.5, 0.87)*	*1.16 (0.93, 1.45)*	*0.99 (0.74, 1.33)*	*0.89 (0.67, 1.18)*	ADT + BPs + Doc	**1.43 (0.83, 2.53)**	**1.4 (0.88, 2.19)**	NA	NA
*1.06 (0.85, 1.33)*	*0.73 (0.57, 0.95)*	*0.774 (0.6, 0.99)*	*0.554 (0.4, 0.75)*	*0.97 (0.75, 1.26)*	*0.834 (0.6, 1.14)*	*0.75 (0.55, 1.03)*	*0.84 (0.62, 1.13)*	ADT + CEL	**0.98 (0.62, 1.53)**	NA	NA
*1.14 (1.01, 1.29)*	*0.79 (0.67, 0.93)*	*0.83 (0.7, 0.97)*	*0.59 (0.47, 0.76)*	*1.05 (0.88, 1.24)*	*0.89 (0.69, 1.15)*	*0.80 (0.63, 1.03)*	*0.9 (0.72, 1.13)*	*1.07 (0.83, 1.39)*	ADT + BPs	NA	NA
*1.28 (1.05, 1.57)*	*0.88 (0.7, 1.12)*	*0.93 (0.74, 1.17)*	*0.67 (0.5, 0.89)*	*1.17 (0.93, 1.49)*	*1 (0.74, 1.36)*	*0.90 (0.67, 1.21)*	*1.01 (0.77, 1.33)*	*1.2 (0.89, 1.63)*	*1.12 (0.89, 1.42)*	ADT + BIC	NA
*0.96 (0.72, 1.28)*	*0.66 (0.49, 0.91)*	*0.7 (0.51, 0.95)*	*0.5 (0.35, 0.71)*	*0.88 (0.64, 1.2)*	*0.75 (0.52, 1.09)*	*0.68 (0.47, 0.97)*	*0.76 (0.54, 1.07)*	*0.91 (0.62, 1.31)*	*0.84 (0.61, 1.15)*	*0.75 (0.61, 0.92)*	ADT + FTA

AAP, abiraterone + prednisone; Doc, docetaxel; ENZA, enzalutamide; APA, apalutamide; RT, radiotherapy; BPs, bisphosphonate; CEL, celecoxib; BIC, bicalutamide; FTA, flutamide; ADT, androgen-deprivation therapy; NA, not applicable.

## Data Availability

The datasets used and analyzed during the current study are available from the corresponding author upon request.
